# In Vitro and In Vivo Biological Activities of *Cissus adnata* (Roxb.)

**DOI:** 10.3390/biomedicines5040063

**Published:** 2017-10-30

**Authors:** Mohammed Shoibe, Md. Nazim Uddin Chy, Morshed Alam, Md. Adnan, Md. Zobidul Islam, Shababa Wajida Nihar, Nishat Rahman, Ehsan Suez

**Affiliations:** 1 Pharmacognosy and Phytochemistry Lab, Department of Pharmacy, International Islamic University Chittagong, Chittagong 4318, Bangladesh; mohammedshoibe48@gmail.com (M.S.); morshedalam3070@gmail.com (M.A.); mdadnan1991.pharma@gmail.com (M.A.); mdzobidulislam@gmail.com (M.Z.I.); 2 Drug Discovery, GUSTO A Research Group, Chittagong 4000, Bangladesh; shababa0037@gmail.com (S.W.N.); nishatrahman.oshin.bd@gmail.com (N.R.); 3 Department of Pharmacy, BGC Trust University Bangladesh, Chittagong 4000, Bangladesh; 4 Department of Biotechnology and Genetic Engineering, Jahangirnagar University, Savar, Dhaka 1342, Bangladesh; ehsansuez666666@gmail.com

**Keywords:** polyphenol, antioxidant, cytotoxic, antibacterial, anthelmintic, antinociceptive, *Cissus adnata*

## Abstract

This study was conducted to evaluate the in vitro polyphenol content, antioxidant, cytotoxic, antibacterial, anthelmintic properties, and in vivo antinociceptive activity of the ethanol extract of *Cissus adnata* leaves (EECA) in different experimental models. Polyphenol contents were investigated using spectrophotometric techniques. Antioxidant activity was determined by 1,1-diphenyl-2-picrylhydrazyl radical (DPPH) radical-scavenging, ferric reducing power, and total antioxidant capacity assays. Cytotoxicity was determined by brine shrimp lethality bioassay and disc diffusion method was used for the antibacterial activity. Anthelmintic activity was studied using aquarium worm (*Tubifex tubifex*) whereas antinociceptive activity was evaluated in mice by acetic acid and formalin test. Phytochemical screening of EECA revealed the presence of alkaloids, carbohydrates, flavonoids, phenols, terpenoids, saponins, and tannins. EECA showed strong antioxidant activity with high polyphenol contents. It was observed that EECA possessed significant antibacterial activity with a low toxicity profile. EECA also demonstrated dose-dependent and statistically significant anthelmintic and antinociceptive activities. Our study shows that ethanol extract of *C. adnata* leaves possess strong antioxidant, antibacterial, anthelmintic and antinociceptive activities with lower toxicity. Further studies are needed to identify bioactive phytomolecules and to understand the mechanism of such actions better.

## 1. Introduction

*Cissus adnata* (Roxb.), a member of Vitaceae family, is a deciduous, large woody climber with yellow flowers, popularly known as Isswarmuli (Chakma), Mach toi kitab (Tripura), Alianga lota and Bhatia lota (Bengali). It is widely distributed and naturally grown at Khagrachari, Bandarban, Cox’s Bazar, Sylhet and Chittagong regions in Bangladesh [1]. Different parts of this plant are used in traditional medicine to treat a variety of diseases and disorders. The leaf of the plant is traditionally used for the treatment of boils, buboes, sores, wounds, stomach upset, peptic ulcer, syphilis, snake bites, urolithiasis, rheumatic pain, bone fractures, and painful menstruation [1,2,3,4]. Stems are used for jaundice and paralysis [1,5]; the root of the plant is used as a diuretic, blood purifier, and also for the treatment of elephantiasis [1,2]. Besides, the plant is also beneficial to treat scurvy, cancer, hemorrhoids, dysentery, epilepsy, fever, asthma, malaria, and food poisoning [2,3].

Preliminary phytochemical screening revealed that the plant contains α- and β-amyrins, β-sitosterol, ketosteroids, carotene, vitamin C, tannins, and phenols [3]. Moreover, three phytochemicals have been isolated from the leaf part such as triterpenoid, flavonoid, and apigenin [6]. Pharmacological studies of the whole plant showed that the plant possesses antioxidant (DPPH free radical scavenging activity), cytotoxic and membrane stabilizing properties [3].

Even though the leaves of *C. adnata* have important medicinal values, so far, no studies have examined its polyphenol content, antioxidant, cytotoxic, antibacterial, anthelmintic and antinociceptive properties. Therefore, the objective of the present study was to evaluate the polyphenol content, antioxidant, cytotoxic, antibacterial, anthelmintic and antinociceptive activities of the ethanol leaf extract of *C. adnata* (EECA) in different experimental models for the first time.

## 2. Materials and Methods

### 2.1. Drugs and Chemicals

All drugs and chemicals used in this research were of analytical grade. Ethanol, methanol, acetic acid, hydrochloric acid, ferric chloride, potassium ferricyanide, phosphate buffer, sodium carbonate, formalin, dimethyl sulfoxide (DMSO) were purchased from Merck ((Darmstadt, Germany). 1,1-diphenyl-2-picrylhydrazyl radical (DPPH), Folin-Ciocalteau reagent (FCR), vanillin, gallic acid, and trichloroacetic acid were obtained from Sigma Chemicals Co. (St. Louis, MO, USA). Diclofenac sodium (Square Pharmaceuticals Ltd., Dhaka, Bangladesh); vincristine sulfate (Techno Drugs Ltd., Dhaka, Bangladesh); levamisole (ACI Limited, Sonargaon, Bangladesh); Ascorbic acid, quercetin, and catechin (BDH Chemicals Ltd. Poole, UK) were procured from the mentioned sources.

### 2.2. Experimental Animals

Swiss albino mice (both sex) weighing approximately 25–30 g were used for this experiment. The mice were collected from Jahangir Nagar University, Savar (Manikganj Highway), Dhaka 1342, Bangladesh. The test animals were maintained standard laboratory conditions (room temperature 25 ± 2 °C; 55–60% relative humidity; 12 h light/dark cycle) and provided with standard laboratory food and distilled water ad libitum. All the experiments were conducted in noiseless condition, and the animals were acclimatized to laboratory conditions for 10 days prior to experimentation.

### 2.3. Ethical Statement for Using Experimental Animals

The experimental animals were conducted according to the guidelines of National Institutes of Health (NIH) and International Council for Laboratory Animal Science (ICLAS) which are internationally acceptable for proper use of laboratory animals. According to the guidelines of NIH and ICLAS, the current study protocol (Pharm-P&D-61/08′14-121, 5 September 2016) was reviewed and approved by the “Ethical review committee” and also by the “Planning and Development Committee” of the Department of Pharmacy, International Islamic University Chittagong, Bangladesh.

### 2.4. Plant Material Collection and Identification

Fresh leaves of *Cissus adnata* (Roxb.) were collected from Bhatiary, Chittagong (22°22′N 91°48′E), Bangladesh in October 2014. The plant sample was authenticated by Professor Dr. Shaikh Bokhtear Uddin, a botanist at the University of Chittagong, Chittagong 4331, Bangladesh. A voucher specimen sample has been deposited in the Herbarium of the University of Chittagong for future reference under the number 7605 CTGUH.

### 2.5. Preparations of Ethanol Extract

The collected fresh leaves were washed, cut into small parts, dried in the shade and finally ground into coarse powder. The powdered plant material (about 450 g) was taken in a clean, flat-bottomed glass container and soaked in 800 mL of 95% ethanol. The glass container with its contents was clogged and retained for a period of 14 days with frequent stirring and shaking. The entire mixture was filtered by a piece of clean and white sterilized cotton materials followed by Whatman No.1 filter paper. Finally, the filtrate solution was evaporated to yield the crude ethanolic extract of *C. adnata* (EECA: 17 g) which was then kept in a refrigerator at 4 °C until further use.

### 2.6. Phytochemical Analysis of EECA

#### 2.6.1. Qualitative Phytochemical Screening

The qualitative phytochemical screening was carried out to check for the presence of different phytochemical constituents like alkaloids, carbohydrates, flavonoids, phenols, tannins, saponins, proteins, amino acid, glycosides, and terpenoids by using standard phytochemical procedures previously described [7].

#### 2.6.2. Quantitative Phytochemical Analysis

##### Determination of Total Phenolic Content

The total phenol content of EECA was evaluated with the Folin-Ciocalteau method [8]. Here, the test samples containing polyphenols are reduced by the Folin-Ciocalteau reagent (FCR) thereby producing a blue colored complex. Briefly, 1 mL (1 mg/mL) of each sample was mixed with 2.5 mL of Folin Ciocalteu’s reagent (previously diluted with water 1:10 *v*/*v*) and 2.5 mL of sodium carbonate (75 g/L) solution. The mixture was shaken for 15 s and allowed to stand for 30 min at 25 °C for color development. Absorbance was then measured at 765 nm against distilled water as a blank by UV-Vis Spectrophotometer (UVmini-1240, Shimadzu, Japan). Gallic acid was used to construct a standard calibration curve. The experiment was conducted in triplicates, and the total content of phenolic compounds was calculated as mg/g gallic acid equivalent (GAE) using the formula: A = (C × V)/m; where A is the total phenolic content (mg/g plant extract in GAE); C is the concentration of gallic acid found from the calibration curve (mg/mL); V is the volume of extract (mL) and m is the weight (g) of the pure plant extract.

##### Determination of Total Flavonoid Content

Total flavonoid content of EECA was evaluated with the previously described method [9]. In short, 1 mL of plant extract (1 mg/mL) or standard of various concentrations was taken in a test tube, and 3 mL of methanol was added. Then 0.2 mL of 10% aluminum chloride solution was added to the same test tube followed by the addition of 0.2 mL of 1 M potassium acetate. Finally, 5.6 mL of distilled water was mixed with the reaction mixture. The reaction mixture was then incubated for 30 min at room temperature to complete the reaction. Then the absorbance of the solution was measured at 415 nm using a UV-Vis Spectrophotometer (UVmini-1240, Shimadzu, Japan) against a blank (methanol served as blank). The experiment was conducted in triplicates, and the total flavonoid content was expressed in mg/g quercetin equivalent (QE) of the plant extract.

##### Determination of Total Flavonols Content

Total flavonol content of the plant extract (EECA) was evaluated with the method previously described by Kumaran and Karunakaran [10], using quercetin (QE) as standard. The reaction mixture consisted of 2 mL of sample, 2 mL of AlCl_3_ prepared in ethanol and 3 mL of sodium acetate solution (50 g/L). After 2.5 h of incubation at 20 °C, the absorbance was measured at 440 nm using a spectrophotometer (UVmini-1240, Shimadzu, Japan). The experiment was conducted in triplicates, and the total flavonol content was expressed as quercetin equivalent (QE)/g of the dried plant extract. For the quercetin, the calibration curve was established using the equation: *y* = 0.0255*x*, R*^2^* = 0.9812, where *x* is the absorbance and *y* is the quercetin equivalent.

##### Determination of Total Condensed Tannins Content

Total condensed tannin or proanthocyanidin content of the plant extract (EECA) was determined according to the method described by Sun et al. [11]. Briefly, the extracts (0.5 mL, 1 mg/mL) were mixed with 3 mL of 4% vanillin-methanol solution (*v*/*v*) and 1.5 mL of hydrochloric acid and slightly vortexed the whole mixture. Then, the mixture was allowed to stand for 15 min at room temperature, and the absorbance was measured at 500 nm using Spectrophotometer (UVmini-1240, Shimadzu, Japan). The experiment was conducted in triplicate, and the total proanthocyanidin content was evaluated at a concentration of 0.1 mg/mL and expressed as catechin equivalent (mg/g) using the calibration curve equation: *y* = 0.5825*x*, R^2^ = 0.9277, where x and y are the absorbance and catechin equivalent respectively.

### 2.7. Acute Oral Toxicity Test of EECA

To evaluate the safety of EECA, acute oral toxicity study was carried out according to OECD Guidelines [12] for the Testing of Chemicals (Test No. 420: Acute Oral Toxicity). Swiss albino mice (both sex) weighing approximately 25–30 g were used for acute oral toxicity test and kept in the experimental room for at least 5 days prior to the start of dosing to allow for acclimatization to the standard laboratory conditions. The experimental animals were kept fasting overnight prior to administration of the oral dose. Then each group of mice (*n* = 6) received a single oral dose (5, 50, 300, and 2000 mg/kg) of the EECA and additionally, the supply of food was withheld for 3 to 4 h. After receiving the doses, the mice were observed for a possible change of behavioral parameters (convulsion, hyperactivity, sedation, and grooming), allergic reactions (itching and skin rash) and mortality for the next 72 h.

### 2.8. In Vitro Antioxidant Activity of EECA

#### 2.8.1. DPPH Free Radical Scavenging Activity

The free radical scavenging activity of the plant extract (EECA) was carried out in terms of hydrogen donating or radical-scavenging ability using the stable radical DPPH (1,1 Diphenyl-1-picrylhydrazyl) by the method described by Braca et al. [13]. Briefly, 0.1 mL of extract at various concentrations (3.125–100 μg/mL) was mixed with 3 mL of freshly prepared DPPH solution (0.004%) in methanol. The mixture was homogenized and incubated for 30 min at room temperature in the dark. The absorbance was measured at 517 nm in a spectrophotometer (UVmini-1240, Shimadzu, Japan). Ascorbic acid was used as the antioxidant standards; methanol used as a blank; methanol plus DPPH solution used as a negative control (A_c_). The degree of decolorization of DPPH from purple to yellow indicated the scavenging efficiency of the extract. All experiment was conducted in triplicates. The percentage of free radical-scavenging activity was expressed with the following formula: Scavenging effect (%) = [(A_c_ − A_s_)/A_c_] × 100. Where A_c_ is the absorbance of the control, and A_s_ is the absorbance of the sample (ascorbic acid or extract). The IC_50_ (half-maximal inhibitory concentration) was calculated graphically using a calibration curve in the linear range by plotting the extract concentration versus the corresponding scavenging effect.

#### 2.8.2. Ferric Reducing Power Capacity (FRP)

The reducing power of the plant extract (EECA) was evaluated according to the method of Oyaizu [14]. Briefly, 1 mL of test solutions at different concentrations (7.81–500 μg/mL) were mixed with 2.5 mL of phosphate buffer (0.2 M, pH 6.6) and 2.5 mL of 1% *w*/*v* potassium ferricyanide. The reaction mixture was incubated for 20 min at 50 °C. After incubation, 2.5 mL of 10% trichloroacetic acid solution was added, and the mixture was centrifuged at 3000 rpm for 10 min. Then, 5 mL of the upper layer solution was mixed with 5 mL of distilled water and 1 mL of 0.1% ferric chloride solution (*w*/*v*), and the absorbance was read at 700 nm in a spectrophotometer (UVmini-1240, Shimadzu, Japan) against a blank sample. Ascorbic acid was taken as a reference standard. The assay was conducted in triplicates, and the results are expressed as mean ± standard error mean (SEM). The increase in absorbance of the sample with concentrations indicates the high reducing potential of the samples.

#### 2.8.3. Determination of Total Antioxidant Capacity (TAC)

Total antioxidant activity of the plant extract (EECA) was determined by the phosphomolybdate method [15], using ascorbic acid as standard. The phosphomolybdate assay is based on the reduction of Mo (VI) to Mo (V) by the plant extract and subsequent formation of a green phosphate/Mo (V) complex at acidic pH. Briefly, 0.3 mL of plant extract was mixed with 3 mL of reagent solution (0.6 M sulfuric acid, 28 mM sodium phosphate and 4 mM ammonium molybdate). The tubes containing the reaction solution were incubated for 90 min at 95 °C. After the samples had cooled to room temperature, the absorbance of the solution was measured at 695 nm using a spectrophotometer (UVmini-1240, Shimadzu, Japan) against a blank (0.3 mL of methanol in the place of the plant extract used as the blank). Ascorbic acid equivalents were calculated using the standard graph of ascorbic acid. The experiment was conducted in triplicate, and calculated values are expressed as equivalent of ascorbic acid in mg/g of dried plant extract.

### 2.9. In Vitro Cytotoxicity Screening of EECA

Brine shrimp lethality bioassay is a widely used method for screening the cytotoxic properties of bioactive compounds; here the method used was similar to that previously reported by Meyer et al. [16]. The eggs of the brine shrimp (*Artemia salina*, a simple zoological organism) were collected from an aquarium shop and hatched in artificial seawater (3.8% NaCl in distill water) at a temperature of about 37 °C with constant oxygen supply for 48 h to mature the shrimps called nauplii. In this study, the test samples of EECA were prepared by dissolving them in DMSO (not more than 50 μL of 5 mL solution) to attain concentrations of 15, 25, 50, 100, 150, 250, 500, 800 and 1000 μg/mL in 5 mL artificial seawater with 10 nauplii in each test tube. A test tube containing 50 μL DMSO diluted to 5 mL artificial seawater was used as a negative control and vincristine sulfate was used as a positive control at concentrations of 0.312, 0.625, 1.25, 2.5, and 5 μg/mL. After 24 h incubation periods, the number of surviving nauplii in each test tube was counted by using a magnifying glass. From the collected data, the percent (%) of lethality of the brine shrimp nauplii was calculated for each concentration using the following formula:

(1) $$\%\;\mathrm{Mortality}=\frac{N_{t}}{N_{0}}\times 100$$

where, *N_t_* = Number of dead nauplii after 24 h of incubation; *N*_0_ = Number of total nauplii transferred (*n* = 10) and the median lethal concentration (LC_50_) was then determined.

### 2.10. In Vitro Antibacterial Activity of EECA

#### 2.10.1. Test Organisms Used in This Study

Three bacterial strains of Gram-positive (*Bacillus cereus*, *Bacillus subtilis*, and *Staphylococcus aureus*), and four strains of Gram-negative bacteria (*Salmonella typhi*, *Salmonella paratyphi*, *Escherichia coli*, and *Pseudomonas aeruginosa*) were used to assess the antibacterial effect of EECA. All of these organisms were sub-cultured in nutrient agar media. They were collected from the Department of Pharmacy, International Islamic University Chittagong, Chittagong 4318, Bangladesh.

#### 2.10.2. Disc Diffusion Assay

Disc diffusion assay (DDA) is a widely accepted method for the evaluation of antimicrobial activity [17]. In DDA method, the plant sample or an antibiotic was diffused from a confined source through the nutrient agar medium, and a concentration gradient was created. The dry, sterilized filter paper discs 6 mm in diameter (Whatman No.1 filter paper, Bibby RE200, Sterilin Ltd., London, UK) containing the known concentration of the test samples (800, and 1000 μg/disc) were placed on nutrient agar media consistently seeded with the test organisms. As positive control standard antibacterial drug kanamycin (30 μg/disc) was used, and blank discs were used as a negative control. For the maximum diffusion of the test materials to the surrounding media, these plates were reserved at low temperature (4 °C) for 12 to 18 h. The plates were then incubated at 37 °C for 24 h to allow optimum growth of the microbes. If the test materials have antibacterial activity, it will inhibit the growth of microorganism having a clear, distinct zone called “Zone of Inhibition”. The antibacterial activity of the test samples is determined by measuring the diameter of the zone of inhibition in term of mm.

### 2.11. In Vitro Anthelmintic Activity of EECA

The anthelmintic activity was conducted according to the method described by Ajaiyeoba et al. [18] with some modifications. In the present study, an aquarium worm (*Tubifex tubifex*) was used to perform the anthelmintic test because of its anatomical and physiological resemblance with an intestinal worm, i.e., Annelida. The aquarium worms were collected from the local market of Chittagong, and worms with average size of 2 to 2.5 cm in length were used for the experiment. Here, the experiment was conducted in triplicate and randomly divided into five groups: Group I served as negative control used only distill water, Group II served as positive control used standard drug levamisole (1 mg/mL), Groups III, IV and V served as test groups at three different concentrations of EECA (5, 8 and 10 mg/mL) respectively. In short, approximately 10 to 12 worms were taken in each petri dish in five groups, and 3 mL of extract solution (EECA) of different concentrations were added. Then the starting time, time of paralysis and time of the death of the worms were noted carefully. The anthelmintic activity was determined at two different stage ‘time of paralysis’ and ‘time of death’ of the worms. The paralyzing time was noted when no movement of any sort could be observed except that the worms were shaken vigorously. After confirming that the worms moved neither when vigorously shaken nor when dipped in slightly warm water, the time of death was recorded.

### 2.12. In Vivo Analgesic Activity of EECA

#### 2.12.1. Experimental Design

Experimental animals were randomly divided into four groups and each group consisted of six mice (*n* = 6). In this study, the test groups received EECA at the doses of 200 and 400 mg/kg while positive control was treated with diclofenac sodium (10 mg/kg) and control with vehicle (1% Tween-80 in distilled water).

#### 2.12.2. Acetic Acid-Induced Writhing Test in Mice

The acetic acid-induced writhing test was performed according to a previously reported method [19] with some modifications for the evaluation of the analgesic potential of EECA. In this study, Group I was administered 1% Tween-80 in distill water which served as control, Group II was administered standard drug diclofenac sodium (10 mg/kg) while groups III and IV were administered with EECA viz., 200 and 400 mg/kg respectively. After 30 min, 0.6% acetic acid (10 mL/kg) was injected intraperitoneally (i.p) into the test models (mice). The number of writhing (contraction of the abdomen, twisting of the mice trunk, elongation, an extension of body and limbs) was counted from 5 to 30 min after the administration of acetic acid. Analgesic activity was expressed as percent of writhing inhibition (%) and calculated by using the following formula:

% inhibition = [(Wc − Wts)/Wc] × 100(2)

where, Wc is the mean number of writhing of the control group, and Wts is the mean number of writhing of the test sample.

#### 2.12.3. Formalin-Induced Licking Test in Mice

The formalin-induced licking test was performed according to a previously reported method with some modifications [20]. After 30 min, 20 μL of 2.5% (*v*/*v* in distilled water) formalin was injected subcutaneously into the plantar surface of the right hind paw of the mice. In this study, Group I was administered 1% Tween-80 in distill water which served as control, Group II was administered standard drug diclofenac sodium (10 mg/kg) while groups III and IV were administered with EECA viz., 200 and 400 mg/kg respectively. The formalin-induced licking of the paw was considered as indicative of the nociceptive behavior. The total time spent in the behavioral responses to nociception including licking and biting of injected paw was recorded. The total time spent was recorded up to 30 min where the first 5 min was considered as early phase (neurogenic phase) and the second period of 15–30 min as the late phase (inflammatory phase) of the nociceptive response. Analgesic activity was expressed as the percentage inhibition of licking time and calculated by using the following formula:

% inhibition = [(Lc − Lts)/Lc] × 100(3)

where, Lc is the licking time of the control group in seconds, and Lts is the licking time of the test sample in seconds.

### 2.13. Statistical Analysis

Calculated values are expressed as mean ± SEM. Data analysis among the groups was compared using one-way ANOVA followed by Dunnett’s test where *p* value < 0.05 was considered to be statistically significant. The statistical analysis was performed in this study by using SPSS software (Statistical Package for Social Science, version 16.0, IBM Corporation, Armonk, NY, USA).

## 3. Results

### 3.1. Qualitative and Quantitative Phytochemical Screening

In order to determine the types of phytoconstituents present in the EECA, the qualitative phytochemical test was performed according to conventional methods, and the results obtained from these experiments are summarized in Table 1. The analyses revealed the presence of alkaloids, carbohydrates, flavonoids, phenols, terpenoids, saponins, and tannins. The total phenol, flavonoid, and condensed tannins content of EECA were 249.33 ± 0.79 mg GAE/g, 24.22 ± 0.35 mg QE/g and 26.41 ± 1.35 mg CA/g dried extract, respectively (Table 2). On the other hand, the total flavonols content of EECA was expressed in Quercetin (QE), and the result was represented in Table 2 where the total flavonols content was 19.35 ± 1.16 mg QE/g dried plant extract.

### 3.2. Acute Toxicity Test

No mortality and behavioral change were recorded at the specified doses during the 72 h observation period of acute toxicity study. So, the dose up to 2000 mg/kg was considered as safe for *C. adnata*, and the median lethal dose (LD_50_) of the EECA was found to be greater than 2000 mg/kg.

### 3.3. In Vitro Antioxidant Activity

#### 3.3.1. DPPH Free Radical Scavenging Activity

**Figure 1:**
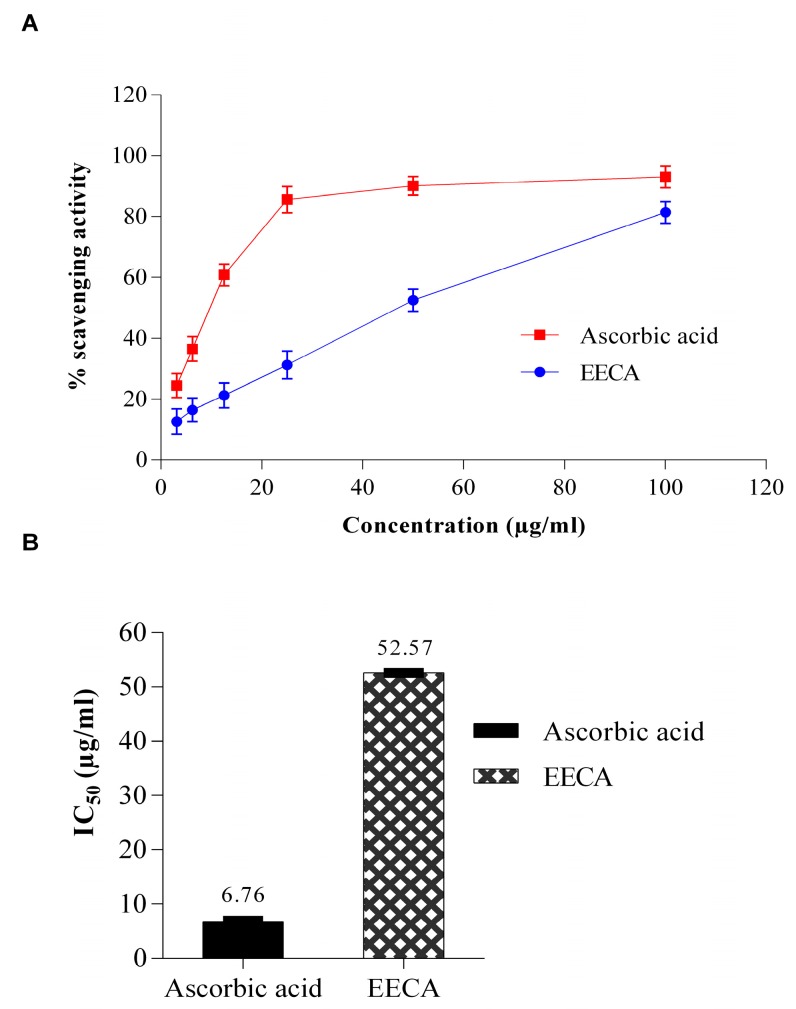
1,1-diphenyl-2-picrylhydrazyl radical (DPPH) free radical scavenging activity of ethanol extract of *C. adnata* leaf (EECA) compared with the standard as assessed by spectrophotometric method using DPPH free radicals. (**A**) Percentage (%) of DPPH radical scavenging by different concentrations of the EECA and reference standard Ascorbic acid. Results are represented as mean ± SD (*n* = 3); (**B**) IC_50_ for DPPH radical scavenging activity of the EECA and Ascorbic acid.

Free radical scavenging activity of EECA was measured by DPPH method as shown in Figure 1. Percentage of scavenging activity was plotted against log concentration and from the graph IC_50_ (Inhibition concentration 50) value was calculated by linear regression analysis. The EECA exhibited significant DPPH free radical scavenging effects compared to standard ascorbic acid. Where the IC_50_ value of ascorbic acid was 6.76 μg/mL and EECA was 52.57 μg/mL.

#### 3.3.2. Ferric Reducing Power Capacity

**Figure 2:**
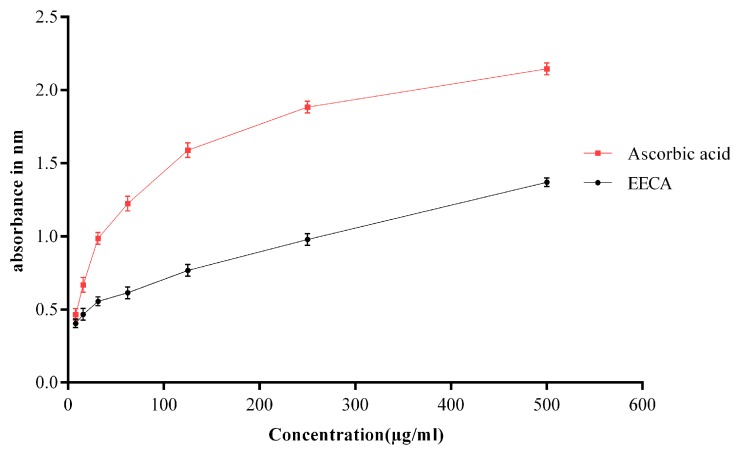
Reducing power capacity of EECA compared with the reference standard ascorbic acid.

The reducing power of a compound is related to its electron transferability and may, therefore; serve as an indicator of its potential antioxidant activity. Data for the reducing powers of EECA was shown in Figure 2. A dose-dependent reducing capability was observed in reducing power assay.

#### 3.3.3. Total Antioxidant Activity

The total antioxidant capacity of EECA was expressed in Ascorbic acid (AA) equivalents and the result was represented in Table 2 where the total antioxidant capacity was 124.80 ± 1.08 mg AA/g dried plant extract.

### 3.4. In Vitro Cytotoxicity Screening

**Figure 3:**
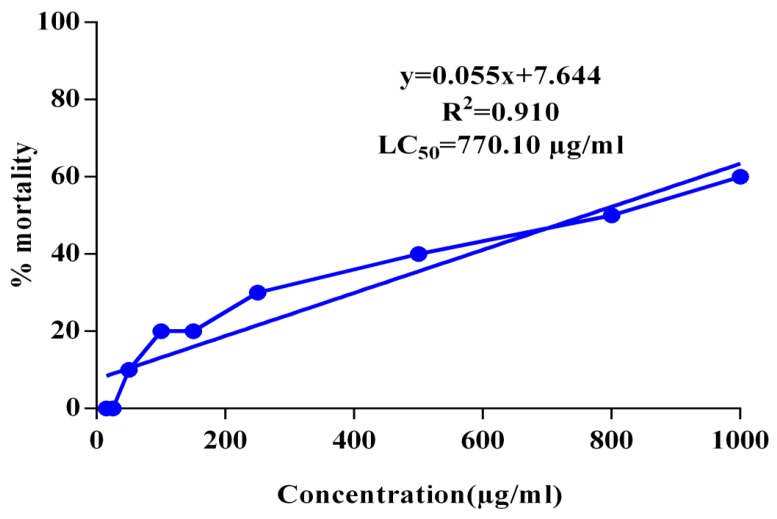
Determination of LC_50_ value of ethanol extract of the *Cissus adnata* leaf from linear correlation between concentrations versus percentage of mortality.

Brine shrimps lethality assay was used to check the cytotoxic effect of the EECA in different concentrations (15 to 1000 μg/mL) that have been presented in Figure 3. The positive control (vincristine sulfate) and negative control (using DMSO and Sea water) were also used to compare the toxic activities of the extract. The LC_50_ value for the ethanol extract of *C. adnata* leaf was found to be 770.10 μg/mL, and that of vincristine sulfate was 0.89 μg/mL. However, no mortality was obtained for the negative control group.

### 3.5. In Vitro Antibacterial Activity

The antibacterial activities of the ethanol extract of *C. adnata* leave obtained by the disc diffusion method are presented in Table 3. The extracts showed different zones of inhibition at three different concentrations (800, and 1000 μg/disc) against three gram-positive and four gram-negative bacteria. The test sample exerted better activity at 800, and 1000 μg/disc concentration against the following tested microorganisms (*Bacillus cereus*, *Salmonella typhi*, *Salmonella paratyphi*, and *Pseudomonas aeruginosa).* The maximum zone of inhibition was obtained against *Bacillus cereus* (13.56 mm). EECA showed better antibacterial activity against gram-negative bacteria than gram-positive. However, the extracts exhibited no activity against three microorganisms, namely *Staphylococcus aureus*, *Bacillus subtilis*, *Escherichia coli*.

### 3.6. In Vitro Anthelmintic Activity

Following the procedure of Ajaiyeoba, the anthelmintic activity of ethanol extract of *C. adnata* leaf (EECA) was determined on an aquarium worm (*Tubifex tubifex*). The degree of anthelmintic activity shown by the extracts was found to be directly proportional to the concentration of the extract ranging from the lowest concentration (5 mg/mL) to the highest concentration (10 mg/mL). At the concentrations of 5 mg/mL, 8 mg/mL, and 10 mg/mL, the ethanolic extract showed significant paralysis time of 32.89, 15.61, 8.10 min and significant death time of 90.66, 55.12 and 29.36 min respectively (Table 4). These results were compared to that of the standard drug of Levamisole at 1 mg/mL concentration which showed a paralysis time of 3.22 min and death time of 6.19 min.

### 3.7. In Vivo Antinociceptive Activity

#### 3.7.1. Acetic Acid Test

**Figure 4:**
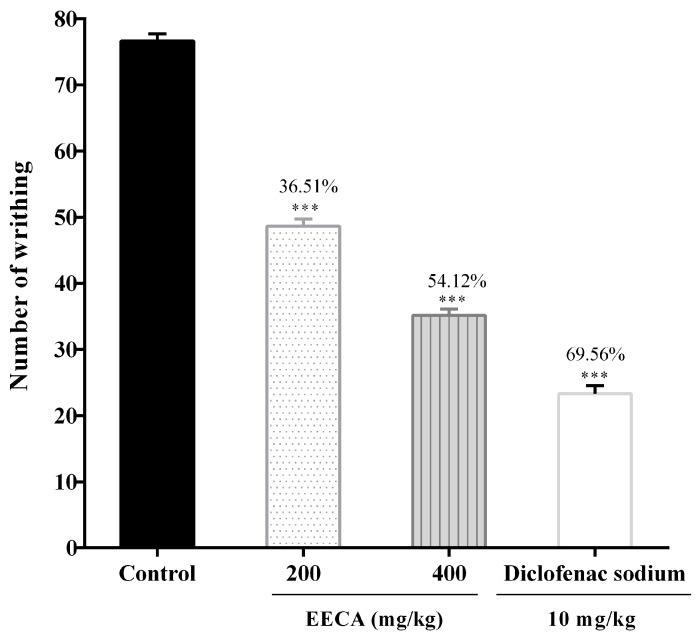
Antinociceptive effect of ethanol extract of *Cissus adnata* (EECA) in acetic acid-induced abdominal writhing test in mice. Each value is presented as mean ± SEM (*n* = 6). *** *p* ˂ 0.001 compared with the control group (Dunnett’s Test).

The effect of ethanol extract of *C. adnata* leaves on acetic acid-induced writhing test in Swiss albino mice administered in a dose-dependent fashion significantly (*p* < 0.001) decreased the number of writhing movements induced by the intraperitoneal administration of the acetic acid compared with the positive control (Figure 4). The highest inhibition of pain exhibited by higher dose 400 mg/kg of EECA was 54.12% while that of the standard drug (Diclofenac sodium, 10 mg/kg) was 69.56%.

#### 3.7.2. Formalin Test

**Figure 5:**
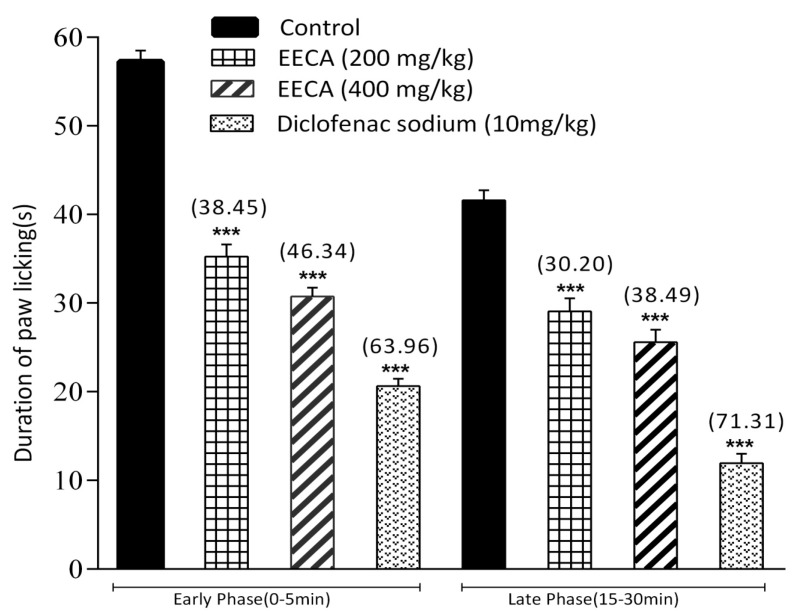
Antinociceptive effect of ethanol extract of *Cissus adnata* (EECA) in formalin-induced licking test in mice. Each value is presented as mean ± SEM (*n* = 6). *** *p* ˂ 0.001 compared with the control group (Dunnett’s Test).

The effect of the extract of *C. adnata* on formalin-induced pain in mice is shown in Figure 5. The extract significantly (*p* < 0.001) inhibited the licking response in both the early phase (38.45% at 200 mg/kg and 46.34% at 400 mg/kg) and the late phase (30.20% at 200 mg/kg and 38.49% at 400 mg/kg) of the formalin test which were comparable to those of the standard drug (Figure 4). Both of these inhibitions were in a dose-dependent manner.

## 4. Discussion

The preliminary phytochemical analysis conducted on ethanol leaf extract of *Cissus adnata* (EECA) revealed the presence of alkaloids, carbohydrates, flavonoids, phenols, terpenoids, saponins, and tannins. In addition, the acute toxicity study showed no occurrence of death or abnormal behavior up to a 2000 mg/kg dose for EECA, which indicates that the extract possesses a low toxicity profile.

Free radicals are known to play a major role in a wide variety of pathological manifestations [21]. This becomes notorious when produced in excess in a living body and causes oxidative damage. Furthermore, it destroys the immunity system of the body and develops a wide range of diseases such as atherosclerosis, angina pectoris, Alzheimer’s disease, Parkinson’s disease, complication in diabetes, rheumatoid arthritis, cardiovascular disease, liver diseases, renal failure, DNA damage, cancer, aging, metabolic disorders, etc. [9,22,23,24]. In this regard, antioxidants can play a vital role in which they fight against free radicals and protect us from various diseases. They exert their action either by scavenging the reactive oxygen species or protecting the antioxidant defense mechanisms [21]. For the experimental evaluation of antioxidant activity of EECA, we began our investigation with the DPPH test which is a widely used technique to evaluate the free radical scavenging activity of plant extracts. This test is based on the reduction of the ethanolic DPPH solution in the presence of antioxidant resulting in the formation of nonradical DPPH-H by the reaction. The stable DPPH were reduced by the plant extracts, and therefore the color changes from purple to yellow to varying degree depending on the presence of antioxidant compounds. The degree of discoloration indicates the scavenging potential of the test sample [25]. In the current study, the EECA exhibited strong antioxidant potential against DPPH free radicals, as shown in Figure 1.The DPPH free radical scavenging activity of EECA increased with increasing concentration. Several bioactive phytochemical components, especially phenolic compounds (flavonoids, phenolic acids, and tannins) are very important components for the free radical scavenging effect as well as antioxidant activities of plants [26,27]. Besides, polyphenols are generally of the chemical patterns; phenolic groups react as hydrogen donors and neutralize the free radicals [27]. The free radical scavenging activity of the extract might be due to the presence of such polyphenolic compounds. Secondly, we performed reducing power assay to measure the antioxidant capacity of EECA. In reducing power assay, the color of the test solution changes to yellow from green depending on the reducing capacity of the test solution. The presence of the reductants in the solution causes the reduction of the Fe^3+^ to Fe^2+^. So, Fe^2+^ can be monitored by absorbance measurement at 700 nm. Recent studies suggested that the reducing properties have been shown to exert antioxidant action by donating of a hydrogen atom to break the free radical chain [28]. The antioxidants present in the EECA caused their reduction of Fe^3+^ to Fe^2+^ and thus proved the reducing power. Like the DPPH free radical scavenging activity, the reducing power of EECA increased with increasing concentration. The ferric reducing power activity of the extract seems to be due to the presence of polyphenolic compounds, and it may serve as a significant indicator of its potential antioxidant activity [29]. The previous report stated that the plant extract that has reducing power could prevent liver injury by inhibiting the formation of lipid peroxides [30]. Thirdly, the total antioxidant capacity of the EECA was measured using the phospho-molybdenum method, based on the reduction of Mo (VI) to Mo (V) by the plant extract and subsequent formation of a green phosphate-Mo (V) complex at acidic pH with a maximum absorption at 695 nm. The present study demonstrated that EECA showed the strong antioxidant capacity for phosphomolybdate reduction. This antioxidant capacity may be the presence of flavonoid and polyphenolic compounds since an earlier report suggested that polyphenolics and flavonoids contribute significantly to the phosphomolybdate scavenging activity of medicinal plants [31,32].

Brine shrimp lethality is a general bioassay which indicates cytotoxicity as well as a wide range of pharmacological activities such as antibacterial, pesticidal, antiviral, antitumor, etc., of the compounds and plant extracts [16]. In this study, various concentrations of EECA ranging from 15 to 1000 μg/mL were used to determine its cytotoxicity as shown in Figure 3. The LC_50_ value for the crude ethanol extract was found to be very high suggesting that the plant extract is safe at the therapeutic doses. Furthermore, researchers found a correlation between the presence of secondary plant metabolites and cytotoxic effects of plant constituents [33]. This correlation is also consistent with our current investigation since our preliminary qualitative, and quantitative phytochemicals analysis of the EECA revealed the occurrence of alkaloids, flavonoids, and tannins which have anti-cancer properties [34,35].

For the development of antimicrobial drugs, plant-derived natural products are important sources of potentially useful structures, because they are natural, easily available, and more profitable [36]; therefore, antibacterial activity assay is the preliminary step towards this goal. Acceptance of medicines from such plant origins as an alternative form of healthcare is increasing day by day because they are serving as promising sources of novel antibiotic prototypes. Additionally, these compounds may have different mechanisms of action than conventional drugs and could be of clinical importance to improve health care [37]. The relatively wide range of antibacterial properties for the extract can be explained by the presence of different kinds of potentially active secondary metabolites detected in them. Indeed flavonoids, phenols, tannins, terpenoids, and alkaloids, identified in the EECA during phytochemical analysis have been reported to possess antimicrobial activities [38,39]. The antibacterial effect of EECA may be due to an individual or a combined effects of the identified groups of phytoconstituents. Our present study justifies the claimed uses of *C. adnata* in the traditional medicines to treat various infectious diseases caused by the microbes through further chemical and pharmacological investigations is essential to isolate and identify potent bioactive molecules that are responsible for this effect.

An infection of parasitic helminths is a major problem in human and animals that cause a chronic and debilitating disease which ultimately leads to death [39,40]. In this regard, traditional medicines have been considered as a great source of easily available effective anthelmintic agents to the peoples of developing country since various medicinal plants have reported previously to possess significant anthelmintic activity [39,41]. Besides, natural sources such as plants can give new biologically active agents that have no or fewer side effects than synthetic drugs and more importantly they are compatible with human physiology [42]. In this study, observations were made for the time taken for paralysis and death of individual worms against the crude extract and the reference drug, levamisole. Here the reference drug, levamisole works as a nicotinic acetylcholine receptor agonist, and it causes persistent stimulation of the parasitic worm muscles, leading to paralysis and ultimately leads to death [43]. The present study concludes that the plant under investigation has been found to possess significant anthelmintic activity in a dose-dependent fashion. This may be described by the fact that several bioactive phytoconstituents such as alkaloids [40], tannins [44], flavonoids [45], and phenolics [45] were found predominantly during qualitative phytochemical screening of the EECA and also a considerable amount of phenol (249.33 ± 0.79 mg GAE/g), flavonoids (24.22 ± 0.35 mg QE/g), and condensed tannins (26.41 ± 1.35 mg CA/g) content have been found in EECA which have significant anthelmintic potential. The phytochemicals, namely alkaloids [40], have the ability to produce paralysis by acting on CNS while polyphenols and tannins selectively bind to free proteins present in the gastrointestinal tract and eventually cause mortality [45]. The anthelmintic efficacy of the EECA may be due to a single compound or combined effect of these phytochemicals.

The first test to assess the antinociceptive activity of EECA was the acetic acid-induced writhing test. The intraperitoneal administration of acetic acid causes the release of endogenous substances like prostaglandins (PGs), histamine, serotonin, bradykinin, cyclooxygenase (COX), and cytokines. These visceral inflammatory mediators enter the dorsal horn of central nervous system and stimulate primary afferent nociceptors and result in the induction of pain expressing abdominal constriction [46]. Oral administration of EECA significantly (*p* < 0.001) reduced the number of abdominal constrictions induced by acetic acid in mice (Figure 4). The result clearly suggests that the antinociception produced by EECA is due to its inhibition of COX, prostaglandins and other endogenous inflammatory mediators. We have detected the presence of carbohydrates, flavonoids, alkaloids, and tannins by preliminary phytochemical screening and a significant amount of polyphenols content has been found in *C. adnata* leaf extract. Scientific investigations reported that plant materials containing flavonoids, phenols, terpenoids, and tannins are responsible for central nervous system and analgesic activity [46,47]. Furthermore, several categories of flavonoids and alkaloids have been found to be anti-inflammatory and antinociceptive agents due to their ability to block arachidonic acid metabolism. Therefore, it is possible that the presence of flavonoids and alkaloids in EECA may be responsible for the antinociceptive potential [47]. A positive result with this test is an indication of antinociceptive activity in the extract, even though it remains to be determined whether this activity is of central or peripheral origin.

To differentiate between the central and peripheral antinociceptive activity of EECA, the formalin test was performed. The formalin-induced licking test is a valid model used in pain and analgesia research and has been reported to produce distinct biphasic (neurogenic and inflammatory phase) nociceptive responses. The early phase (0 to 5 min) is believed to be caused by non-inflammatory pain resulting from direct stimulation of nociceptors and the late phase (15 to 30 min) is considered as an inflammation-induced pain that is linked with inflammatory cytokines (like serotonin, histamine, bradykinin, and prostaglandins) in the periphery and activation of the neurons in the dorsal horns of the spinal cord [48]. In general, both phases have their own characteristics that can be used as a tool to assess the antinociceptive effect as well as to explain the mechanisms of antinociception. It has been reported that centrally acting drugs (i.e., narcotics/opioids like morphine, heroin, etc.) inhibit both phases of the formalin test, whereas peripherally acting drugs (i.e., NSAIDs such as such as aspirin, diclofenac sodium, indomethacin, dexamethasone, etc.) inhibit only the late phase [49]. Results of the present study show that the plant extract produced antinociception against both the neurogenic and inflammatory phase of formalin induction. Therefore, the results shown by EECA suggest that the extract possesses both central and peripheral antinociceptive effects and additional anti-inflammatory activity. These types of activity might be due to the presence of flavonoids, alkaloids, and terpenoids since the previous report suggested that these bioactive phytoconstituents are responsible for significant analgesic and anti-inflammatory activities [47].

Plant materials rich in polyphenols are the major plant compounds with antioxidant activity [29]. This activity is exhibited mainly because of their redox properties, which play an important role in absorbing and neutralizing free radicals. Polyphenols are also very important because their hydroxyl groups provide scavenging ability [27]. There are several categories of polyphenols like flavonoids, phenolic acids, flavonols, stilbenes, tannins, lignans, etc. Chief among them are the flavonoids, which have strong antioxidant activities [50]. Besides, flavonoids are naturally occurring in plants and are thought to have positive effects on human health. The previous report on flavonoid derivatives has shown a wide range of antibacterial, antinociceptive, anti-inflammatory, anticancer, antiviral, and anti-allergic activities [51,52]. Flavonoids have also been shown to be highly effective scavengers of most oxidizing molecules, including quenching singlet and triplet oxygen or decomposing peroxides, and various free radicals implicated in several diseases [53]. Tannins are known to be beneficial in the treatment of inflamed or ulcerated tissues, and they have extremely good activity in most cancers prevention [9]. Therefore, comparable with the findings in the literature for other extracts of plant products, our results suggested that alkaloids, phenol, flavonoids, terpenoids, and tannins may be the major contributors to the antioxidant activity, free radical scavenging activity, cytotoxicity, antibacterial, anthelmintic, and antinociceptive activities of ethanol leaf extract of *C. adnata* (EECA).

## 5. Conclusions

In a nutshell, the results of the current study demonstrate that the ethanol leaf extract of *C. adnata*, may be due to its polyphenol contents, which showed strong antioxidant, antibacterial, anthelmintic and antinociceptive activities with lower toxicity. However, further extensive research on chemical characterization of the plant needs to be performed to find the presence of most bioactive phytomolecules and to know the underlying mechanism behind the pharmacological activity.

## Figures and Tables

**Table 1:** The result of preliminary phytochemical screening of ethanol leaf extract of *Cissus adnata*.

Name of the Phytochemicals	EECA
Alkaloids	+
Carbohydrates	+
Flavonoids	+
Phenols	+
Tannins	+
Saponins	+
Proteins	−
Amino acid	−
Glycosides	−
Terpenoids	+

EECA denotes for ethanol extract of *Cissus adnata*; (+) = present and (−) = absent.

**Table 2:** Total phenolics, flavonoids, flavonols, condensed tannins content and total antioxidant capacity of ethanol leaf extract of *Cissus adnata*.

Tested Extract	Total Phenol Content (mg GA/g DE)	Total Flavonoid Content (mg QE/g DE)	Total Flavonols Content (mg QE/g DE)	Total Condensed Tannins Content (mg CA/g DE)	Total Antioxidant Capacity (mg AA/g DE)
EECA	249.33 ± 0.79	24.22 ± 0.35	19.35 ± 1.16	26.41 ± 1.35	124.80 ± 1.08

Each value in the table is represented as mean ± SEM (*n* = 3). EECA refers to ethanol extract of *Cissus adnata*; GA, gallic acid; QE, quercetin; CA, catechin; AA, Ascorbic acid; DE, dried extract.

**Table 3:** Antibacterial effects of ethanol extract of *Cissus adnata* leaves.

Name of the Bacteria	Zone of Inhibition (mm)		
	Kanamycin Disc (30 μg/disc)	EECA (μg/disc)	
		800	1000
**Gram-positive**			
*Staphylococcus aureus*	29.30 ± 0.60	-	-
*Bacillus subtilis*	25.29 ± 0.35	-	-
*Bacillus cereus*	27.50 ± 0.58	8.60 ± 0.50	13.56 ± 0.51
**Gram-negative**			
*Salmonella typhi*	28.18 ± 0.81	9.16 ± 0.76	11.76 ± 0.40
*Salmonella paratyphi*	30.51 ± 0.50	9.51 ± 0.50	12.63 ± 0.85
*Escherichia coli*	31.20 ± 0.82	-	-
*Pseudomonas aeruginosa*	25.46 ± 0.70	6.33 ± 0.57	10.40 ± 0.53

EECA refers to ethanol extract of *Cissus adnata*; Values are mean inhibition zone (mm) ± SD of three replicates; -: no activity.

**Table 4:** Anthelmintic activity of ethanol leaf extract of *Cissus adnata*.

Treatment	Time is Taken for Paralysis (min)	Time is Taken for Death (min)
NC (Water)	0.00	0.00
PC (1 mg/mL)	3.22 ± 0.05	6.19 ± 0.35
EECA (10 mg/mL)	8.10 ± 0.66 ***	29.36 ± 0.98 ***
EECA (8 mg/mL)	15.61 ± 1.03 ***	55.12 ± 1.49 ***
EECA (5 mg/mL)	32.89 ± 0.75 ***	90.66 ± 2.18 ***

Each value in the table is represented as mean ± SEM (*n* = 3); EECA denotes for ethanol extract of *Cissus adnata*; NC, Negative control; PC, Positive control (Levamisole). *** *p* < 0.001 compared with positive control group (Dunnett’s test).

## Abbreviations

EECA	Ethanol extract of *Cissus adnata*
ANOVA	Analysis of variance
SEM	Standard error mean
Fe^2+^	Ferrous
Fe^3+^	Ferric
SPSS	Statistical Package for the Social Sciences
i.p	Intraperitoneal
